# Novel approaches to the treatment of type 1 diabetes

**DOI:** 10.1111/1753-0407.13333

**Published:** 2022-11-07

**Authors:** Zachary Bloomgarden

**Affiliations:** ^1^ Endocrinology, Diabetes and Bone Disease Icahn School of Medicine at Mount Sinai New York New York USA

Type 1 diabetes (T1D) is an immune‐mediated illness, with selective destruction of β‐cells occurring in genetically predisposed persons.^1^ In addition to apoptotic β‐cell destruction after immune damage, a reduction in β‐cell proliferative capacity may play a role in determining which individuals go on to develop T1D. In the present commentary, we discuss several novel approaches that may prove useful in early treatment of T1D. Complex immunomodulatory treatments prior to the onset of clinical disease have been shown to reduce the risk of developing T1D. Such approaches, however, require the identification of very small numbers of predisposed persons in the overall population, a daunting exercise. A German study carried out in more than 150 000 children found only 0.35% to be positive for islet antibodies, of whom only 0.08% progressed to develop diabetes.^2^ The identification of a number of experimental treatment options for T1D after clinical onset offer intriguing alternative possibilities.

Serologic markers such as antibodies to islet cells and glutamic acid decarboxylase‐65 are typically present prior to clinical presentation of disease and lend themselves to measurement, contributing to T1D by presenting islet antigens, including insulin, leading to activation of T cells causing autoimmune destruction of pancreatic beta cells.^3^ These islet antigens are expressed in the thymus during development, normally promoting development of T cell‐mediated immune tolerance^4^; most β‐cell destruction is mediated by autoreactive T‐cells. Most approaches to preventing T1D have been directed at modulation of the autoimmune response. Human leukocyte antigen (HLA) class II allele DQ8 forms unstable complexes with β‐cell constituents such as insulin expressed in the thymus during development leading to decreased presentation of these antigens with consequent escape of autoreactive T cells from thymic negative selection. As a result, activation of autoreactive T cells in the periphery subsequently in life can initiate the autoimmune process causing T1D.^5^ Some 50%–60% of persons with T1D have the HLA‐DR4/DQ8 haplotype, with the presence of DQ8 associated with a 6.5‐11‐fold increase in T1D risk. DQ8 is thought to present insulin and proinsulin peptides leading to activation of CD4+ helper T lymphocytes, in turn stimulating other immune cells, such as macrophages, B lymphocytes, and CD8+ T lymphocytes. By analysis of the molecular configuration of DQ8 and insulin peptides, it was found that these form hydrogen bonds to DQ8 at four sites. A small molecule, the antihypertensive agent methyldopa, blocks this binding. A recent phase 1 trial in 20 HLA DQ8 positive persons with recent onset T1D showed that administration of methyldopa was associated with reduction in insulin‐specific CD4+ T cells, with reduction in HbA1cwith C‐peptide levels not declining.^6^, ^7^

The gut microbiome has been studied in a number of areas in medicine. Of interest, microbial fermentation of nondigestible carbohydrates can lead to production of several short chain fatty acids, including acetate, which is then taken up by pancreatic lymph nodes, where it appears to decrease autoreactive T cells as well as proinflammatory cytokines such as interleukin‐21, and butyrate, which increases regulatory T‐cell numbers,^8^ with the potential to further reduce islet inflammation and apoptosis.^9^ Activation of the endogenous gut cannabinoid system may be another approach to increase pancreatic lymph node regulatory T‐cell activity.^10^ There has been interest in vitamin D as a therapeutic approach, with several trials of vitamin D and vitamin D analog administration suggesting potential benefit in early T1D,^11^ although other trials have been negative and the duration of improvement in insulin secretion with such approaches appears to be limited.^12^

An entirely different treatment of T1D has addressed antiapoptotic/pro‐proliferative agents. Paired box gene 4 (PAX4) is a transcription factor involved in differentiation of the endoderm‐derived endocrine pancreas, which appears to be reactivated to promote islet cell growth in response to immune destruction. Individuals homozygous for the PAX4 C allele have reduced PAX4 function in comparison to those heterozygous for PAX4 A and PAX4 C, or to PAX4 A homozygotes. In several population studies, approximately three quarters of persons with T1D expressed PAX4 C/C, whereas this genotype was present in approximately one third of controls. The PAX4 C/C genotype was present in only 11% of a small group of persons positive for markers of islet autoimmunity who did not develop T1D over a long period of observation.^13^

Thioredoxins are antioxidant and anti‐inflammatory proteins present in all organisms, with thioredoxin‐interacting protein present in β‐cells blocking the action of thioredoxin, leading to activation of the inflammasome and playing roles in β‐cell glucotoxicity and apoptosis. The calcium channel blocker verapamil is another small molecule antihypertensive agent that has been found to reduce β‐cell cytoplasmic levels of ionized calcium, in turn inhibiting thioredoxin‐interacting protein. In a study of 24 persons with new‐onset T1D treated with verapamil, preservation of C‐peptide, reduction in insulin dosage requirement, and reduction in levels of the β‐cell autoantigen chromogranin A at 1 year^14^ and at 2 years^15^ were demonstrated. Interestingly, administration of R‐verapamil to individuals with type 2 diabetes has been shown to reduce levels of HbA1c,^16^ suggesting that the mechanism of action of verapamil may be related to reduction in β‐cell apoptosis and/or to β‐cell proliferation rather than to an immunomodulatory effect.

A potentially related approach to increasing β‐cell proliferation involves inhibition of dual specificity tyrosine‐regulated kinase 1A (DYRK1A), which blocks β‐cell proliferation caused by calcium entering the β‐cell via voltage‐dependent calcium channels.^17^ A number of DYRK1A inhibitors have been found to induce β‐cell proliferation in vitro.^18^ A small molecule plant alkaloid derived from L‐tryptophan, harmine, is one such DYRK1A inhibitor, investigated in the past for central nervous system effects and now being considered as an agent to increase endogenous insulin secretory capacity.^19^ A small molecule glucagon‐like peptide 1 receptor agonist is also being studied as a potential agent to reduce pancreatic β‐cell apoptosis and dysfunction.^20^

Thus, it appears that a number of lines of investigation suggest approaches that may prove of benefit in the treatment of early T1D. It is intriguing to speculate that some of these agents may be effective in combination, with, for example, both verapamil and harmine altering cytoplasmic ionized calcium levels or with combination of a pro‐proliferative agent one reducing β‐cell autoimmunity. We look forward to the results of ongoing clinical trials.
